# Supplementary material for: Modeling glioblastoma heterogeneity as a dynamic network of cell states
Source: Mol Syst Biol. 2021 Sep 16;17(9):e10105. doi: 10.15252/msb.202010105 (PMC8444284; doi:10.15252/msb.202010105)
Supplement: Supplementary file 6 — Source Data for Figure 5 [file MSB-17-e10105-s004.zip › Figure5A_sourcedata/GSEA_3017/nosigdb_stateA.GseaPreranked.1622795026485/DOROSZKO_U3180MG_CLUSTER_3.html]

Details for gene set DOROSZKO\_U3180MG\_CLUSTER\_3[GSEA]

|  || Dataset | state53017 |
| Phenotype | NoPhenotypeAvailable |
| Upregulated in class | na\_pos |
| GeneSet | DOROSZKO\_U3180MG\_CLUSTER\_3 |
| Enrichment Score (ES) | 0.44938233 |
| Normalized Enrichment Score (NES) | 3.6717255 |
| Nominal p-value | 0.0 |
| FDR q-value | 0.0 |
| FWER p-Value | 0.0 |
Table: GSEA Results Summary

  

Fig 1: Enrichment plot: DOROSZKO\_U3180MG\_CLUSTER\_3      
 Profile of the Running ES Score & Positions of GeneSet Members on the Rank Ordered List

  

| PROBE | GENE SYMBOL | GENE\_TITLE | RANK IN GENE LIST | RANK METRIC SCORE | RUNNING ES | CORE ENRICHMENT || 1 | CDK1 |  |  | 3 | 1.006 | 0.0145 | Yes |
| 2 | MKI67 |  |  | 4 | 0.926 | 0.0311 | Yes |
| 3 | UBE2C |  |  | 7 | 0.793 | 0.0429 | Yes |
| 4 | MCM10 |  |  | 9 | 0.752 | 0.0552 | Yes |
| 5 | BUB3 |  |  | 12 | 0.740 | 0.0661 | Yes |
| 6 | KPNA2 |  |  | 20 | 0.667 | 0.0698 | Yes |
| 7 | HIST1H4C |  |  | 21 | 0.659 | 0.0816 | Yes |
| 8 | ASPM |  |  | 27 | 0.639 | 0.0871 | Yes |
| 9 | PCLAF |  |  | 28 | 0.637 | 0.0985 | Yes |
| 10 | DNAJC9 |  |  | 34 | 0.615 | 0.1036 | Yes |
| 11 | PGAM1 |  |  | 35 | 0.608 | 0.1145 | Yes |
| 12 | CKS2 |  |  | 40 | 0.587 | 0.1203 | Yes |
| 13 | NCAPG |  |  | 44 | 0.556 | 0.1267 | Yes |
| 14 | HJURP |  |  | 47 | 0.539 | 0.1340 | Yes |
| 15 | CKS1B |  |  | 50 | 0.530 | 0.1411 | Yes |
| 16 | NUDT5 |  |  | 52 | 0.528 | 0.1494 | Yes |
| 17 | DBF4 |  |  | 55 | 0.514 | 0.1563 | Yes |
| 18 | SMC3 |  |  | 58 | 0.504 | 0.1629 | Yes |
| 19 | MASTL |  |  | 60 | 0.499 | 0.1707 | Yes |
| 20 | MIS18BP1 |  |  | 64 | 0.495 | 0.1760 | Yes |
| 21 | CCNA2 |  |  | 65 | 0.495 | 0.1849 | Yes |
| 22 | TPX2 |  |  | 66 | 0.488 | 0.1936 | Yes |
| 23 | MYBL2 |  |  | 69 | 0.486 | 0.1999 | Yes |
| 24 | HNRNPF |  |  | 70 | 0.485 | 0.2086 | Yes |
| 25 | SGO2 |  |  | 73 | 0.478 | 0.2148 | Yes |
| 26 | VRK1 |  |  | 82 | 0.470 | 0.2138 | Yes |
| 27 | UBE2T |  |  | 83 | 0.470 | 0.2222 | Yes |
| 28 | DUT |  |  | 87 | 0.466 | 0.2270 | Yes |
| 29 | TUBB4B |  |  | 93 | 0.462 | 0.2294 | Yes |
| 30 | CENPF |  |  | 94 | 0.461 | 0.2376 | Yes |
| 31 | TOP2A |  |  | 95 | 0.457 | 0.2458 | Yes |
| 32 | HIST1H1D |  |  | 98 | 0.456 | 0.2516 | Yes |
| 33 | PRTFDC1 |  |  | 104 | 0.448 | 0.2537 | Yes |
| 34 | ZWINT |  |  | 106 | 0.447 | 0.2605 | Yes |
| 35 | AURKB |  |  | 108 | 0.446 | 0.2674 | Yes |
| 36 | KIF23 |  |  | 116 | 0.436 | 0.2669 | Yes |
| 37 | DEPDC1 |  |  | 119 | 0.432 | 0.2723 | Yes |
| 38 | MCMBP |  |  | 122 | 0.428 | 0.2776 | Yes |
| 39 | MELK |  |  | 123 | 0.427 | 0.2852 | Yes |
| 40 | SGO1 |  |  | 126 | 0.425 | 0.2905 | Yes |
| 41 | BUB1B |  |  | 129 | 0.421 | 0.2956 | Yes |
| 42 | KNL1 |  |  | 133 | 0.418 | 0.2996 | Yes |
| 43 | HELLS |  |  | 135 | 0.417 | 0.3059 | Yes |
| 44 | RTKN2 |  |  | 141 | 0.413 | 0.3074 | Yes |
| 45 | PIMREG |  |  | 143 | 0.413 | 0.3136 | Yes |
| 46 | GTSE1 |  |  | 146 | 0.411 | 0.3186 | Yes |
| 47 | MAD2L1 |  |  | 153 | 0.406 | 0.3188 | Yes |
| 48 | KIFC1 |  |  | 160 | 0.402 | 0.3189 | Yes |
| 49 | ARHGAP11A |  |  | 162 | 0.399 | 0.3248 | Yes |
| 50 | GNG4 |  |  | 164 | 0.398 | 0.3308 | Yes |
| 51 | KIF2C |  |  | 165 | 0.397 | 0.3379 | Yes |
| 52 | MCM7 |  |  | 171 | 0.394 | 0.3391 | Yes |
| 53 | NDC80 |  |  | 173 | 0.393 | 0.3449 | Yes |
| 54 | CKLF |  |  | 183 | 0.388 | 0.3412 | Yes |
| 55 | METRN |  |  | 186 | 0.387 | 0.3458 | Yes |
| 56 | CMSS1 |  |  | 191 | 0.381 | 0.3479 | Yes |
| 57 | KIF22 |  |  | 197 | 0.377 | 0.3487 | Yes |
| 58 | CDCA3 |  |  | 203 | 0.372 | 0.3495 | Yes |
| 59 | TRIP13 |  |  | 209 | 0.370 | 0.3502 | Yes |
| 60 | SMC4 |  |  | 217 | 0.367 | 0.3485 | Yes |
| 61 | SPC25 |  |  | 226 | 0.363 | 0.3456 | Yes |
| 62 | NCAPH |  |  | 232 | 0.359 | 0.3461 | Yes |
| 63 | H2AFZ |  |  | 245 | 0.351 | 0.3382 | Yes |
| 64 | PPIF |  |  | 248 | 0.350 | 0.3421 | Yes |
| 65 | FEN1 |  |  | 259 | 0.343 | 0.3364 | Yes |
| 66 | TUBB |  |  | 264 | 0.340 | 0.3378 | Yes |
| 67 | CENPJ |  |  | 270 | 0.339 | 0.3379 | Yes |
| 68 | LRR1 |  |  | 273 | 0.337 | 0.3416 | Yes |
| 69 | CLSPN |  |  | 274 | 0.336 | 0.3476 | Yes |
| 70 | DEPDC1B |  |  | 275 | 0.335 | 0.3536 | Yes |
| 71 | ESCO2 |  |  | 282 | 0.333 | 0.3525 | Yes |
| 72 | ORC6 |  |  | 283 | 0.333 | 0.3585 | Yes |
| 73 | CACYBP |  |  | 288 | 0.330 | 0.3596 | Yes |
| 74 | C21orf58 |  |  | 294 | 0.328 | 0.3596 | Yes |
| 75 | GGCT |  |  | 298 | 0.326 | 0.3619 | Yes |
| 76 | MND1 |  |  | 304 | 0.324 | 0.3618 | Yes |
| 77 | RAD18 |  |  | 307 | 0.324 | 0.3652 | Yes |
| 78 | BRCA2 |  |  | 308 | 0.323 | 0.3710 | Yes |
| 79 | CENPN |  |  | 310 | 0.323 | 0.3756 | Yes |
| 80 | FANCI |  |  | 319 | 0.319 | 0.3719 | Yes |
| 81 | TUBA1C |  |  | 322 | 0.318 | 0.3752 | Yes |
| 82 | TEDC1 |  |  | 324 | 0.317 | 0.3797 | Yes |
| 83 | RFC3 |  |  | 337 | 0.314 | 0.3712 | Yes |
| 84 | CDCA5 |  |  | 341 | 0.312 | 0.3732 | Yes |
| 85 | CLIC1 |  |  | 342 | 0.311 | 0.3788 | Yes |
| 86 | PRC1 |  |  | 350 | 0.310 | 0.3760 | Yes |
| 87 | ANP32B |  |  | 357 | 0.307 | 0.3744 | Yes |
| 88 | SLC20A1 |  |  | 361 | 0.306 | 0.3764 | Yes |
| 89 | CDCA2 |  |  | 369 | 0.304 | 0.3735 | Yes |
| 90 | BRCA1 |  |  | 373 | 0.302 | 0.3754 | Yes |
| 91 | CKAP2 |  |  | 379 | 0.301 | 0.3749 | Yes |
| 92 | RPA3 |  |  | 381 | 0.300 | 0.3791 | Yes |
| 93 | CSE1L |  |  | 382 | 0.300 | 0.3845 | Yes |
| 94 | RRM2 |  |  | 383 | 0.300 | 0.3898 | Yes |
| 95 | XRCC2 |  |  | 384 | 0.300 | 0.3952 | Yes |
| 96 | CENPW |  |  | 386 | 0.299 | 0.3994 | Yes |
| 97 | TEX30 |  |  | 388 | 0.298 | 0.4035 | Yes |
| 98 | CDC6 |  |  | 391 | 0.297 | 0.4065 | Yes |
| 99 | PHF19 |  |  | 393 | 0.297 | 0.4106 | Yes |
| 100 | HMGA1 |  |  | 397 | 0.295 | 0.4124 | Yes |
| 101 | SAAL1 |  |  | 398 | 0.295 | 0.4177 | Yes |
| 102 | RACGAP1 |  |  | 399 | 0.293 | 0.4229 | Yes |
| 103 | PA2G4 |  |  | 402 | 0.293 | 0.4258 | Yes |
| 104 | NME4 |  |  | 404 | 0.291 | 0.4298 | Yes |
| 105 | CCT5 |  |  | 409 | 0.290 | 0.4303 | Yes |
| 106 | HMGB2 |  |  | 424 | 0.288 | 0.4189 | Yes |
| 107 | POP7 |  |  | 427 | 0.287 | 0.4217 | Yes |
| 108 | NUSAP1 |  |  | 430 | 0.286 | 0.4244 | Yes |
| 109 | NUF2 |  |  | 434 | 0.285 | 0.4260 | Yes |
| 110 | MIS18A |  |  | 439 | 0.284 | 0.4264 | Yes |
| 111 | LMNB1 |  |  | 442 | 0.282 | 0.4291 | Yes |
| 112 | CDC45 |  |  | 446 | 0.282 | 0.4306 | Yes |
| 113 | ANP32E |  |  | 453 | 0.280 | 0.4285 | Yes |
| 114 | PFKP |  |  | 454 | 0.280 | 0.4335 | Yes |
| 115 | GINS1 |  |  | 462 | 0.279 | 0.4302 | Yes |
| 116 | SMS |  |  | 463 | 0.278 | 0.4352 | Yes |
| 117 | MNS1 |  |  | 464 | 0.278 | 0.4402 | Yes |
| 118 | ATAD2 |  |  | 470 | 0.275 | 0.4392 | Yes |
| 119 | EZH2 |  |  | 471 | 0.275 | 0.4441 | Yes |
| 120 | SKA1 |  |  | 475 | 0.274 | 0.4455 | Yes |
| 121 | SHCBP1 |  |  | 477 | 0.274 | 0.4492 | Yes |
| 122 | TRAP1 |  |  | 482 | 0.273 | 0.4494 | Yes |
| 123 | KIF4A |  |  | 504 | 0.269 | 0.4294 | No |
| 124 | NME1 |  |  | 509 | 0.269 | 0.4295 | No |
| 125 | HAUS8 |  |  | 518 | 0.267 | 0.4248 | No |
| 126 | CIP2A |  |  | 522 | 0.266 | 0.4260 | No |
| 127 | PDCD5 |  |  | 526 | 0.265 | 0.4272 | No |
| 128 | RCC1 |  |  | 528 | 0.265 | 0.4308 | No |
| 129 | SKA3 |  |  | 531 | 0.264 | 0.4332 | No |
| 130 | ALYREF |  |  | 535 | 0.264 | 0.4343 | No |
| 131 | RANBP1 |  |  | 536 | 0.263 | 0.4391 | No |
| 132 | KIF15 |  |  | 538 | 0.263 | 0.4426 | No |
| 133 | CDKN3 |  |  | 539 | 0.263 | 0.4473 | No |
| 134 | FBXO5 |  |  | 557 | 0.258 | 0.4318 | No |
| 135 | DDX39A |  |  | 562 | 0.258 | 0.4317 | No |
| 136 | PSMG2 |  |  | 576 | 0.254 | 0.4209 | No |
| 137 | TMPO |  |  | 584 | 0.253 | 0.4171 | No |
| 138 | FAM111A |  |  | 618 | -0.259 | 0.3828 | No |
| 139 | LRRCC1 |  |  | 746 | -0.341 | 0.2388 | No |
| 140 | PEG10 |  |  | 917 | -0.587 | 0.0483 | No |
| 141 | CSRP2 |  |  | 960 | -0.877 | 0.0144 | No |
| 142 | TCEAL9 |  |  | 965 | -0.912 | 0.0260 | No |
Table: GSEA details [plain text format]

  

Fig 2: DOROSZKO\_U3180MG\_CLUSTER\_3: Random ES distribution      
 Gene set null distribution of ES for **DOROSZKO\_U3180MG\_CLUSTER\_3**
